# Predictors of posttraumatic stress symptoms following childbirth

**DOI:** 10.1186/1471-244X-14-200

**Published:** 2014-07-16

**Authors:** Anna N Vossbeck-Elsebusch, Claudia Freisfeld, Thomas Ehring

**Affiliations:** 1 Institute of Psychology, University of Münster, Fliednerstraße 21, 48149 Münster, Germany

**Keywords:** Posttraumatic stress disorder, Childbirth, Delivery, Cognitive factors

## Abstract

**Background:**

Posttraumatic stress disorder (PTSD) following childbirth has gained growing attention in the recent years. Although a number of predictors for PTSD following childbirth have been identified (e.g., history of sexual trauma, emergency caesarean section, low social support), only very few studies have tested predictors derived from current theoretical models of the disorder. This study first aimed to replicate the association of PTSD symptoms after childbirth with predictors identified in earlier research. Second, cognitive predictors derived from Ehlers and Clark’s (2000) model of PTSD were examined.

**Methods:**

*N =* 224 women who had recently given birth completed an online survey. In addition to computing single correlations between PTSD symptom severities and variables of interest, in a hierarchical multiple regression analyses posttraumatic stress symptoms were predicted by (1) prenatal variables, (2) birth-related variables, (3) postnatal social support, and (4) cognitive variables.

**Results:**

Wellbeing during pregnancy and age were the only prenatal variables contributing significantly to the explanation of PTSD symptoms in the first step of the regression analysis. In the second step, the birth-related variables peritraumatic emotions and wellbeing during childbed significantly increased the explanation of variance. Despite showing significant bivariate correlations, social support entered in the third step did not predict PTSD symptom severities over and above the variables included in the first two steps. However, with the exception of peritraumatic dissociation all cognitive variables emerged as powerful predictors and increased the amount of variance explained from 43% to a total amount of 68%.

**Conclusions:**

The findings suggest that the prediction of PTSD following childbirth can be improved by focusing on variables derived from a current theoretical model of the disorder.

## Background

In recent years, posttraumatic stress disorder (PTSD) following childbirth has gained growing attention in research and in the clinical field. This heightened interest was triggered by empirical findings showing that about one third of women who have given birth rate this experience as highly distressing [1,2]. In addition, 25-30% of women experience PTSD symptoms at a subclinical level and/or meet criteria for partial PTSD shortly after having given birth to a child [2,3] and 1.5 to 6% even develop full-blown PTSD [1,4,5]. Qualitative studies have found that apart from experiencing high levels of anxiety and anger, women who develop PTSD after childbirth often also suffer from emotional detachment from their partners and babies as well as from fear of future pregnancy [6-8].

A number of studies have aimed to identify predictors of PTSD following childbirth. This appears relevant not only from a theoretical but also a clinical point of view. Knowledge on predictors of PTSD following this particular event could provide the basis for screening instruments that can be used to identify women in need of psychosocial interventions. In addition, existing postpartum counselling interventions addressing PTSD symptoms following childbirth often use generalized and non-specific strategies and lack empirical foundation [9]. Findings on predictors of post-partum PTSD could therefore guide the development of innovative preventive interventions by shedding the light on key processes that need to be modified.

Past research has focused on three different groups of predictors. First, a number of *prenatal factors* have been found to be associated with PTSD symptoms after childbirth, including giving birth to one’s first child [10,11], a history of sexual trauma [2,12] and difficulties during pregnancy [13]. In addition, being a single parent has been shown to be associated with an elevated level of psychological distress after birth [14].

The second group of predictor variables are *characteristics of the birth experience* itself. Specifically, levels of PTSD were found to be related to concerns for one’s own life or concerns for the baby’s life while giving birth [1,3], emergency caesarean sections [5,15], instrumental delivery [1,16], medical complications concerning the mother or the child after birth [17], levels of negative emotions experienced during birth or shortly after [16], and pain during delivery [2,10,15]. Finally, low social support was found to be associated with PTSD symptoms after childbirth (*postnatal*) [2,3,18], which parallels findings from the literature looking at other types of trauma [19]. Importantly, postnatal social support was found to explain variance in PTSD symptoms over and above other covariates [20].

In sum, a number of prenatal, perinatal and postnatal predictors of symptom levels of PTSD have been identified in the literature until now. However, it is noteworthy that most existing research into PTSD following childbirth is rather atheoretical. Interestingly, studies investigating PTSD following other types of traumatic experiences (e.g., road traffic accidents and physical and/or sexual assault) have shown that the prediction of PTSD can considerably be improved by including variables that are explicitly derived from theoretical models of the disorder. One of the models that has best been supported by empirical evidence is Ehlers and Clark’s cognitive model of PTSD [21-23]. Ehlers and Clark [24] suggest that PTSD symptoms are mainly due to three different processes. First, strong encoding of perceptual information in combination with relatively weak encoding of contextual information is thought to lead to intrusive memories. One commonly studied form of peritraumatic cognitive processing that has been linked to high risk for PTSD symptoms is peritraumatic dissociation. Second, Ehlers and Clark [24] suggest that excessively negative appraisals of the trauma and/or its sequelae are responsible for the development and maintenance of PTSD symptoms. Finally, the model posits that recovery from PTSD is hindered by individuals’ engagement in dysfunctional cognitive coping strategies, including thought suppression and excessive trauma-related rumination. All parts of the model have received considerable empirical support in the general PTSD literature (for a review, see [25]). In order to test whether these findings extend to PTSD following childbirth, the current study included a number of variables derived from Ehlers and Clark’s cognitive model, namely dissociation during childbirth, negative appraisals related to childbirth, and levels of thought suppression and rumination following childbirth.

Three previous studies have tested the role of cognitive factors in the context of PTSD following childbirth. Results show that postpartum levels of PTSD are related to negative appraisals of the birth experience and/or its sequelae [18] as well as dissociation during childbirth [16,26]. However, to our knowledge, up to now no study has tested the model as a whole, including the role of maladaptive cognitive strategies, focusing on postpartum PTSD.

The aims of the current study were threefold. First, we aimed to replicate earlier findings regarding the prediction of PTSD levels following childbirth by known prenatal, perinatal and postnatal predictors. Based on previous findings on PTSD after childbirth, we hypothesized that PTSD symptoms are significantly related to age, partnership status, previous sexual trauma, other previous traumata, previous psychological disorders, wellbeing and complications during pregnancy, giving birth to one’s first child, secondary or emergency caesarean section, instrumental delivery, concern for one’s life or the baby’s life while giving birth, levels of negative emotions experienced during birth, pain during delivery, wellbeing and complications during childbed and low postpartum social support. Second, we tested whether the following variables derived from Ehlers and Clark’s [24] cognitive model are related to PTSD following childbirth: dissociation during childbirth, negative appraisals related to childbirth, and levels of thought suppression and rumination following childbirth. Third, we tested whether the theoretically-derived cognitive variables still significantly predict PTSD symptom levels when the already established predictors of PTSD following childbirth are controlled for.

## Methods

### Sample

The study was conducted as a web-based survey. Inclusion criteria were female gender, having given birth to a child during the last 1 to 6 months and consent to the study. Potential participants meeting these criteria were invited to the study in two ways. First, a network of midwives informed their clients about the study and provided them with the link to the online study. Second, recruitment took place via a selection of German-speaking online fora for mothers.

The survey was started by 521 subjects and 246 (47.21%) completed all questionnaires and repeated their consent at the end of the survey. For ethical reasons, we were only able to retain those participants in the analyses who filled in all questions and repeated their consent to the use of their data for research purposes at the end of the survey^a^. The average time to complete the whole survey was 27 minutes. The following reasons led to the exclusion of 22 women: two women needed less than 10 minutes to fill in the whole survey suggesting possible careless responding, for 17 women the birth was less than four weeks ago, one woman indicated a stillbirth and two women withdrew the consent to the use of their data for research purposes at the end of the survey. The final sample included in the analyses therefore consisted of 224 participants with a mean age of 30.54 (*SD* = 4.56). The majority of participants were married (*n =* 162, 72.3%), one quarter of the participants were in a relationship, but not married (*n =* 58, 25.9%) and four participants were single (1.8%). Most participants (*n =* 156, 69.6%) had experienced a normal vaginal birth, 18 participants (8.0%) had experienced an instrumental delivery, 15 participants (6.7%) had a planned caesarean section, 17 participants (7.6%) had a caesarean section after onset of labour or after bursting of the amniotic sac and 18 participants (8.0%) indicated that they had an emergency caesarean section. According to the regulations of the local ethics committee (Department of Psychology, University of Münster), no ethics approval was needed for this study.

### Instruments

#### Posttraumatic stress disorder

The German version of the Posttraumatic Diagnostic Scale (PDS) [27,28] was used to measure symptom levels of PTSD. The questionnaire contains 49 items and consists of four parts. In the first part, a short checklist identifies potentially traumatizing events experienced which are rated in a “yes”/“no” format. The second part determines with a “yes”/“no” answer format if the A1 (“person experienced, witnessed or was confronted with an event or events that involved actual or threatened death or serious injury, or a threat to the physical integrity of self or others”) and the A2 (“the person’s response involves intense fear, helplessness, or horror”) criteria of the DSM-IV diagnoses are met. The third and main part of the questionnaire contains 17 items which assess re-experiencing, avoidance and numbing symptoms, and hyperarousal during the last month on a 4-point-Likert scale (from “not at all or only one time” to “5 or more times a week/almost always”). The fourth part of the PDS measures impairment in nine different life domains in a “yes”/“no” format. In the current study, participants were instructed to fill in the PDS with regard to the recent childbirth as the index event. As a measure of PTSD symptom severity, the PDS and the German translation have demonstrated high reliability and high correlations with other measures of trauma-related psychopathology [27,28]. To estimate the percentage of probable PTSD, we used an algorithm proposed by Foa et al. [27], which consists of checking (a) whether the individual DSM-IV criteria are fulfilled and (b) whether the overall PDS score is at least 18. DSM-IV criteria of probable PTSD were considered to be present when participants endorsed at least 1 re-experiencing symptom, 3 avoidance or numbing symptoms and 2 arousal symptoms as present and endorsed significant interference with their overall level of functioning. A symptom was scored to be present when participants scored at least 1 (“once a week or less/one in a while”) on the 0–3 response scale of the PDS (compare [29]). This algorithm has shown good sensitivity and validity when compared to the Structural Clinical Interview for DSM-IV Axis I Disorders [29,30]. The German version of the PDS, which we used in our survey, has also been shown to be a reliable and valid measure [28]. In our study, the internal consistency of the 17 items that built the third part of the PDS was *α =* .92.

#### Demographic variables and prenatal risk factors

Demographic variables included gender, age, partnership status (single, married, in a partnership), citizenship, mother tongue and education. Past traumatic experiences were assessed via the first part of the PDS (see above), which provides participants with a list of 12 types of traumatic events and asks them to identify each event that has happened to them. Participants were also asked how many children they have given birth to (including their last childbirth). We also asked whether they have previously been diagnosed with any psychological disorders. Additional potential prenatal risk-factors assessed in the current study were whether participants had experienced any complications during pregnancy, and how they had predominately felt during pregnancy, which they indicated on a 5-point-Likert scale (“very bad*”* to “very good*”*).

#### Birth-related risk factors

In addition to identifying the number of weeks since delivery, participants were also asked to indicate their birth modus. The answer options were normal vaginal birth, planned caesarean section (primary section), caesarean section after onset of labour or after bursting of the amniotic sac (secondary section), emergency caesarean section and vaccum extractor or delivery forceps were used (instrumental delivery). It was only possible to choose one of the answer options concerning birth modus. Moreover, we also assessed the following birth-related variables: child being transferred to a child clinic (“yes”/“no”), complications during childbed (“yes”/“no”), general wellbeing during childbed on a 5-point Likert scale (“very good*”* to “very bad*”*), pain during delivery (scale from 1 to 10) and concern’s for one’s life or the baby’s life during delivery (“yes”/“no”).

Negative emotions during childbirth were measured with a German translation of the Peritraumatic Emotions Questionnaire (PEQ) [31] asking participants to rate the extent to which they had experienced each of 15 different negative emotions during the event (e.g., “humiliated”, “terrified”, “furious”) on a 5-point-Likert scale (“not at all” to “very strongly”). The PEQ has shown a high internal consistency and contributed to the prediction of DSM-IV diagnosis of a PTSD [21,31]. For the German translation used in this study, two researchers independently translated the items into German and a third person translated the items back into English in order to establish the equivalence of the two language versions. In our study, an internal consistency of *α =* .92 emerged.

#### Social support

Social support with an addressed timeframe of the last 4 weeks was assessed with the German short version of the University of California, Los Angeles Social Support Inventory (UCLA-SSI-d) [32,33]. The questionnaire comprises 20 items that cover the areas material aid and assistance (assistance, e.g., “In general, how satisfied or dissatisfied have you been with the assistance you have received in the past month?”), advice or information (advice, e.g., “In general, how satisfied or dissatisfied have you been with all the information and advice you have received in the past month?”) and listening while one expresses beliefs or feelings (emotional support, e.g.,. “In general, how satisfied or dissatisfied have you been with the listening and understanding you have received in the last month?”). The questionnaire assesses on a 5-point Likert scale, how often friends, relatives, partners and medical personnel gave support in these areas (quantity of social support), and it also assesses how satisfied the person was with her social support in the areas advice, assistance and emotional support (quality of social support). In previous studies, the UCLA-SSI has been found to be correlated with birth-outcomes and postpartum depression [34,35]. In our sample, internal consistencies were *α =* .80 for quantity and *α =* .89 for quality of social support.

#### Cognitive variables

Dissociation during childbirth was assessed with the German translation of the Peritraumatic Dissociative Experience Questionnaire (PDEQ) [36,37]. The PDEQ has 10 items (e.g., “What was happening seemed unreal to me, like I was in a dream or watching a movie or play.”), which are rated on a 5-point Likert scale (“not at all true” to “extremely true”) and demonstrated high reliability and convincing validity [36]. In this study, an internal consistency of *α =* .82 was found.

Negative birth-related thoughts and interpretations were assessed with the German version of the Posttraumatic Cognitions Inventory (PTCI) [24,38,39]. The questionnaire comprises 29 items that belong to the subscales negative cognitions about self (18 items, e.g., “I am a weak person*.”*), negative cognitions about the world (6 items, e.g., “You never know who will harm you*”*) and self-blame (5 items, e.g., “Somebody else would have stopped the event from happening*”*). Participants were asked to indicate their answers on a 7-point-Likert scale from “totally disagree*”* to “totally agree*”*. The original and the German version of the PTCI both showed good internal consistencies, and a convincing validity [38,40]. In the current study, for each item of the PTCI containing the word(s) (traumatic) event, the words were replaced by birth. This was done to prevent suggesting that the birth experience was traumatic if the mother did not rate it as traumatic herself and to make sure that the women all referred to the birth experience instead of referring to another trauma that might have occurred previously. In this study, the internal consistency for the scale negative cognitions about self was *α =* .94, for the scale negative cognitions about the word it was *α =* .88, for the scale self-blame it was *α* = .64 and for the sum score for all 29 items it was *α =* .94.

Two subscales of the German version of the Responses to Intrusions Questionnaire (RIQ) were used to assess thought suppression and rumination [41,42]. The RIQ assesses different aspects of responses to intrusive memories. The thought suppression subscale consists of six items (e.g., “I try to erase the memory of the event”). The rumination subscale of the RIQ consists of 7 items (e.g., “I dwell on what I should have done differently”)*.* All items of the RIQ are rated on a 4-point-Likert scale from “Never” to “Always”, indicating the frequency of using each strategy. Again, the questionnaire was slightly modified so that it referred to birth when the original questionnaire referred to a traumatic event. In our study, the internal consistency for the subscale thought suppression was *α* = .90 and for the rumination subscale it was *α = .*89.

We used the German version of the Perseverative Thinking Questionnaire (PTQ) [43] to assess participants’ general tendency to engage in repetitive negative thinking (e.g., in the form of rumination). The PTQ comprises 15 items (e.g., “The same thoughts keep coming to my mind again and again*”*) that are answered on a 5-point Likert scale from “Never” to “Almost Always”. The PTQ has shown a high internal consistency and a good convergent and predictive validity [43] with Cronbach’s *α = .*95 in this study. Different subscores can be computed from the PTQ. However, only the total score was used in the current study as an overall measure of repetitive negative thinking. Excellent psychometric properties have been shown using the PTQ total score [43].

### Statistical analyses

Data analysis was conducted with SPSS, version 22.0. Single associations between predictor variables of interest and PDS scores were computed using Pearson correlation coefficient or point-biserial correlation coefficients for categorical variables. Categorical variables with more than two specifications were analysed with univariate ANOVAs with the PDS score as a dependent variable.

A hierarchical multiple linear regression to predict the PDS score was conducted. In the first step of the hierarchical regression analyses, prenatal variables were entered. In the second step, birth-related variables were added. In the third step, social support was entered. Finally, in a fourth step, the cognitive variables were added. In all steps, only variables showing statistically significant single correlations with the PDS were entered into the regression model. The PDS had a skewness of 1.40 (*SE =* .16) and a curtosis of 1.92 (*SE =* .32). Therefore the PDS was square root-transformed for all analyses. In order to rule out multicollinearity, variance inflation factors were computed. None of the variance inflation factors was > 5 and all tolerance scores were > .25, which means that multicollinearity is negligible [44]. In addition, the Kolmogorov-Smirnov test (*p =* .20) and the Shapiro-Wilk test (*p =* .08) indicated that a normal distribution of the residuals of the regression analysis can be assumed. A significance level of .05 was used for all statistical tests. In the results section, significance always refers to statistical significance. A post-hoc power analysis using the GPower computer program [45] indicated that with the final sample size of 224 and 18 predictors in the hierarchical regression model, assuming a significance level of .05, we achieved a power of 96%.

## Results

### Level of PTSD in the current sample

The participants retained in the analyses showed a mean PDS score of *M =* 8.61 (*SD* = 8.51), with a large range of scores (0–44). When applying the criteria suggested by Foa et al. [27] and Ehring et al. [29], 27 participants (12.05%) showed signs of probable PTSD, two of whom indicated that they had already suffered from these symptoms prior to childbirth. Therefore, a probable PTSD following childbirth was found in 25 participants (11.16%).

### Bivariate correlations of potential predictors with PTSD symptoms

Regarding prenatal variables, age and wellbeing during pregnancy were significantly negatively correlated with the PDS score (see Table 1). Positive associations with PDS scores were found for the dichotomous prenatal variables history of a sexual trauma, history of a non-sexual other trauma and complications during pregnancy (see Table 2). Regarding birth-related variables, negative peritraumatic emotions (PEQ) and complications during childbed both showed a significant positive correlation with the PDS score, whereas wellbeing during childbed was significantly negatively correlated (see Table 1). PDS scores also differed depending on the birth modus, *F*(4, 219) = 7.07, *p* < .001, η_p_^2^ = .11. Post-hoc comparisons using Scheffé tests indicated that the mean PDS score for the emergency caesarean section (*M* = 16.17, *SD* = 11.04, *p =* .001) and for the secondary caesarean section (*M* = 13.53, *SD* = 9.74, *p =* .046) were both significantly higher than the PDS score for women who had a normal vaginal delivery (*M* = 7.04, *SD* = 7.56). All other post hoc comparisons did not reach statistical significance. Therefore, for the regression analysis two dichotomous variables were created to estimate the influence of either a secondary or an emergency section (“yes“/“no“) or a normal vaginal birth (“yes“/“no“).

**Table 1:** Continuous prenatal and birth-related variables and their correlation with PTSD symptoms

**Variable**	***M (SD)***	**Range**	**Correlation with PDS**
Age	30.54 (4.56)	20-42	-.16*
Wellbeing during pregnancy	2.78 (1.08)	1-5	-.21**
Week of delivery	39.71	33-43	-.09
Pain during delivery	7.22 (2.49)	1-10	.06
Peritraumatic emotions (PEQ)	13.41 (12.19)	0-58	.61***
Wellbeing during childbed	2.28 (1.17)	1-5	-.52***
Quantity of social support (UCLA)	52.57 (10.19)	24-75	-.18**
Quality of social support (UCLA)	20.03 (5.61)	4-28	-.25***

*Note. N* = 224. PDS = Posttraumatic Diagnostic Scale, PEQ = Peritraumatic Emotions Questionnaire, SS = Social support, UCLA = University of California, Los Angeles Social Support Inventory.

*p < .05. ** *p <* .01. ***p < .001.

**Table 2:** Dichotomous prenatal and birth related variables and their correlation with PTSD symptoms

**Variable**		**% (*n*)**	**Point-biserial correlation with PDS**
Citizenship German	Yes	96.9 (217)	-.04
Mother tongue German	Yes	97.8 (219)	.01
First Childbirth	Yes	66.1 (148)	.10
Previous Psychological Disorder	Yes	20.5 (46)	.13
History of sexual trauma	Yes	16.5 (37)	.19**
History of other previous trauma	Yes	62.05 (139)	.18*
Concern for one’s life or the baby’s life during childbirth	Yes	25.4 (57)	.34***
Complications during pregnancy	Yes	46.0 (103)	.15*
Complications during childbed	Yes	41.5 (93)	.17*

*Note. N* = 224. PDS = Posttraumatic Diagnostic Scale.

*p < .05. **p < .01. ***p < .001.

Both quality and quantity of postnatal social support showed a significant negative correlation with the PDS score (see Table 1). All cognitive variables were significantly positively correlated with the PDS score (see Table 3). None of the subscales of the PTCI showed a higher correlation with the PDS score than the overall score. Therefore only the overall score was retained in the regression analysis.

**Table 3:** Cognitive variables and their correlation with PTSD symptoms

**Variable**	***M (SD)***	**Range**	**Correlation with PDS**
Dissociation (PEDQ)	10.14 (8.41)	0-41	.44***
PTCI: Overall score	52.60 (26.33)	29-165	.73***
PTCI: Self	28.90 (16.93)	18-106	.73***
PTCI: World	11.52 (7.64)	6-41	.60***
PTCI: Self-Blame	12.18 (6.06)	5-32	.38***
RIQ: Thought Suppression	8.65 (3.66)	6-22	.70***
RIQ: Rumination	10.78 (4.77)	7-28	.76***
PTQ	23.10 (14.11)	0-60	.52***

*Note. N* = 224. PDS = Posttraumatic Diagnostic Scale, PDEQ = Peritraumatic Dissociative Experience Questionnaire, PTCI = Posttraumatic Cognitions Inventory, RIQ = Responses to Intrusions Questionnaire, PTQ = Perseverative Thinking Questionnaire.

*** *p <* .001.

### Hierarchical multiple regression analysis

Results of the hierarchical regression analysis are shown in Table 4. The first model including the prenatal variables history of sexual trauma, history of other previous trauma, complications during pregnancy and wellbeing during pregnancy (Model 1), showed a significant prediction of PDS scores, *R*^*2*^ = .11, *F*(4, 219) = 5.13, *p <* .001. When birth-related variables were additionally included (Model 2), the prediction was significantly improved with an additional 33% of variance in PDS scores accounted for, *R*^*2*^ = .43, *ΔR*^*2*^ = .33; *F*(6, 213) = 20.36, *p <* .001. The inclusion of postnatal social support (Model 3) did not significantly improve the prediction of the PDS score, *ΔR*^*2*^ = .01, *F*(2, 211) = 1.95, *p =* .15. Following inclusion of the cognitive variables in the fourth block (Model 4), the prediction was further improved by a significant amount, *R*^*2*^ = .68, *ΔR*^*2*^ = .24; *F*(5, 206) = 29.61, *p <* .001.

**Table 4:** Hierarchical multiple regression analyses predicting PTSD symptoms from perinatal variables (Model 1), birth-related (Model 2), social support (Model 3) and cognitive variables (Model 4)

**Predictor**	**Model 1**			**Model 2**			**Model 3**			**Model 4**		
	***R***^**2 **^**= .11, Δ *R***^**2 **^= .11***			***R***^**2 **^**= .43, Δ *R***^**2 **^= .33***			***R***^**2 **^**= .44, Δ *R***^**2 **^**= .01**			***R***^**2 **^**= .68, Δ *R***^**2 **^= .23***		
	***B***	***SE B***	***β***	***B***	***SEB***	***β***	***B***	***SE B***	***β***	***B***	***SE B***	***β***
Constant	2.95***			1.54*			2.51**			-.10		
**Block1: prenatal variables**												
Age	-.04	.02	-.13*	-.04	.02	-.11*	-.04	.02	-.11*	-.01	.01	-.04
Previous sexual trauma	.36	.28	.09	.17	.23	.04	.16	.23	.04	.15	.18	.04
Other previous trauma	.36	.22	.11	.13	.18	.04	.10	.18	.03	-.13	.14	-.04
Complications during pregnancy	.34	.22	.11	.09	.18	.03	.10	.18	.04	.09	.14	.03
Wellbeing during pregnancy	-.21	.10	-.15*	-.17	.09	-.12	-.17	.09	-.12	-.08	.07	-.05
**Block 2: birth-related variables**												
Perceived life threat^a^				.11	.23	.03	.14	.23	.04	.26	.18	.07
Peritraumatic Emotions (PEQ)				.04	.01	.35***	.04	.01	.32***	-.01	.01	-.08
Normal Vaginal Birth				-.13	.24	-.04	-.14	.24	-.04	-.22	.19	-.07
Secondary Section or Emergency Section				-.03	.31	-.01	-.01	.30	.00	.04	.24	.01
Complications During Childbed				-.01	.18	.00	.02	.18	.01	.11	.14	.04
Wellbeing During Childbed				-.38	.09	-.29***	-.37	-.09	-.28***	-.21	.07	-.16**
**Block 3: social support**												
Quality of social support (UCLA)							-.02	.02	-.06	-.01	.01	-.01
Quantity of social support (UCLA)							-.01	.01	-.07	-.01	.01	-.03
**Block 4: cognitive variables**												
Dissociation (PDEQ)										.02	.01	.08
PTCI										.01	<.01	.17*
RIQ: Rumination										.07	.02	.22**
RIQ: Thought Supression										.09	.03	.20**
PTQ										.02	.01	.23***

*Note. N =* 224. Total *R*^*2 *^*=* .68. PDS = Posttraumatic Diagnostic Scale – square transformed, PEQ = Peritraumatic Emotions Questionnaire; UCLA = University of California, Los Angeles Social Support Inventory; PDEQ = Peritraumatic Dissociative Experience Questionnaire; PTCI = Posttraumatic Cognitions Inventory; RIQ = Responses to Intrusions Questionnaire; PTQ = Perseverative Thinking Questionnaire.

^a^Concern for one’s life or the baby’s life.

*p < .05. **p < .01. ***p < .001.

## Discussion

The study aimed at analysing the relationship of established predictors of PTSD following childbirth on the one hand and of cognitive variables on the other hand with PTSD symptoms. First, we analysed the bivariate associations and the contributions of each of the established predictors in a regression model to predict PTSD symptoms. Second, we looked at the bivariate associations of the variables derived from the cognitive model of PTSD by Ehlers and Clark. Third, we analysed the contributions of the cognitive variables for the prediction of PTSD symptom levels when the established predictors of PTSD were controlled for.

The first objective of the current study was to replicate the associations of predictors of PTSD symptoms that have been derived from previous research. As expected, on a bivariate level, the prenatal variables age and low wellbeing during pregnancy showed significant negative associations with PTSD symptoms and previous sexual and non-sexual traumata as well as complications during pregnancy were positively related to PTSD symptoms. Importantly, however, only age and wellbeing during pregnancy remained significant in a regression model including all prenatal variables. These findings resemble previous results in the context of PTSD after childbirth [2,12,13] as well as PTSD in general [19,46]. However, unlike previous research on risk factors of PTSD [19,46], we did not find a significant correlation of previous psychological disorders or minority status with PTSD symptoms in our study. Although all prenatal variables taken together significantly predicted PTSD in the first step of the regression analysis, they only accounted for 10% of the variance in PTSD symptoms.

As expected, a number of birth-related variables were also associated with PTSD symptoms on a bivariate level. These included negative peritraumatic emotions, complications during childbed, and general wellbeing during childbed, which are in line with earlier findings [16,17]. Contrary to our expectation and previous findings [2,10,15], pain during delivery did not show a significant association with PTSD symptoms in our sample. In the regression analysis, the addition of birth-related variables increased the amount of explained variance of PTSD symptom severity from 10% to 43%. The only variables that remained significant predictors in the regression model with prenatal and perinatal variables included were age, peritraumatic emotions and wellbeing during childbed. The fact that birth-related variables explain more variance than pre-birth variables parallels findings from meta-analyses on predictors of PTSD in general [19,46], showing that distal risk factors (e.g., prior psychological adjustment, prior history of trauma) show weaker associations with PTSD symptoms than more proximal factors that are directly related to the traumatic event itself (e.g., perceived life threat; peritraumatic emotions).

As expected, low postnatal social support was also positively correlated with PTSD symptoms on a bivariate level. However, our regression analysis showed that it did not significantly contribute to the explanation of variance in PTSD symptom severity over and above pre-birth and birth-related variables. This is surprising as social support has been consistently found to be strongly associated with PTSD symptom severity in previous studies [18,19,46]. However, previous studies have also shown that social support has a stronger explanatory power when a longer period elapses between the potentially traumatic event and data collection [18,19], suggesting that the effects of social support are cumulative over time and/or that social support serves as a secondary prevention strategy [19]. An alternative explanation may be that the influence of social support on PTSD symptoms is mediated by the general wellbeing of the women.

In addition to examining established predictors of PTSD following childbirth, the study aimed to test the role of cognitive variables. We focused on variables derived from Ehlers and Clark’s cognitive model of PTSD [24], as this model has already received a large amount of empirical support from studies following different kinds of trauma (e.g. [21,31,47]). We found that all variables derived from this model (i.e., negative appraisals of the trauma and/or its sequelae, thought suppression, rumination/repetitive negative thinking and peritraumatic dissociation) showed strong positive correlations with PTSD symptom severity on a bivariate level. However, peritraumatic dissociation was not a significant predictor in the regression model including all cognitive variables in addition to other established predictors. Thus, our results replicate the small number of earlier studies showing associations between cognitive variables and PTSD following childbirth. In addition, they extend these earlier findings in important ways. First, whereas earlier studies have investigated single cognitive variables in isolation, we included variables derived from all key processes described by Ehlers and Clark, including maladaptive cognitive coping strategies (thought suppression, rumination and repetitive negative thinking), which have not been investigated in research into PTSD after childbirth, yet. Our result that peritraumatic dissociation is not a significant predictor when other variables are controlled for in the regression model is in line with some earlier findings showing no independent contribution of peritraumatic dissociation [48,49]. Although the metaanalysis of Ozer et al. [19] identified peritraumatic dissociation as one of the strongest predictors of PTSD symptoms, it has been argued that the retrospective assessment of peritraumatic dissociation may be biased [50].

When, we tested whether theoretically-derived cognitive variables improve the statistical prediction of PTSD over and above established risk factors (prenatal, birth-related and social support), results of the regression analysis show that this was indeed the case as all cognitive variables taken together accounted for an additional 23% of variance. These findings show that cognitive variables are important in the development and/or maintenance of postpartum PTSD. Most of the established predictors of PTSD symptoms did not contribute to the prediction of PTSD symptoms once the cognitive variables had been included in the regression model. This is one of the main findings of our study and parallels findings in the context of PTSD following other types of traumas, which also provide evidence for the importance of cognitive variables in the development and maintenance of PTSD [21-23]. In addition, these results suggest that it is promising to focus on variables derived from established theoretical models of PTSD when aiming to identify risk factors for the disorder following childbirth.

Our study shows a number of strengths. These comprise the relatively large sample and the inclusion of different types of risk factors within one multiple regression model. In addition, our study is one of the first studies testing whether an established theoretical model of PTSD is applicable to PTSD cases with onset after childbirth as a particular type of potentially traumatic event. Importantly, cognitive predictor variables not only included negative trauma-related appraisals and dissociation but also dysfunctional cognitive coping strategies (rumination, thought suppression), which are processes that could potentially been targeted in the prevention and/or treatment of PTSD following childbirth.

Apart from these strengths, some limitations warrant mentioning. First, the cross-sectional design of the study makes it impossible to test if the assumed risk factors indeed play a causal role in the development or maintenance of the symptoms. It is possible that the current PTSD symptoms or other factors lead to a retrospective bias in answers to questions regarding the birth experience. Second, the online survey did not allow the establishment of clinical diagnoses. Reassuringly, the algorithm we used to estimate probable PTSD has previously shown good sensitivity and validity when compared to an established structured clinical interview [29]. Nevertheless, a replication of the findings in a study using structured clinical interviews to assess PTSD and other psychological disorders is warranted. As other psychological disorders were assessed by a simple “yes“/“no“ question, some participants might not have been aware that they were suffering from a psychological disorder. In addition, it should be noted that the assessment of PTSD in the current study was still based on the DSM-IV as no validated self-report measure for PTSD according to the DSM-5 [51] was available, yet, at the time of the current study. Third, due to the retrospective design we cannot completely rule out that some of the symptoms were induced by a previous trauma or other previous psychological problems. However, it is of note that previous traumata or previous psychological disorders were not among the statistically significant predictors in the first step of the regression analysis.

Based on the current findings, a number of avenues for future research appear promising. For example, prospective studies are needed testing the predictive power of cognitive variables for postpartum PTSD. In addition, it appears promising to develop and evaluate screening instruments that allow identifying women at risk for PTSD symptoms. The current findings suggest that risk factors such as age, wellbeing during pregnancy, wellbeing during childbed, peritraumatic emotions and/or cognitive variables could be used to develop items for a screener. As many women stay in a mother-child unit during the first days after childbirth [4], this provides a window of opportunity to implement routine screening to identify women in need of early intervention. Another important task for future research arises from the fact that despite the impact of PTSD symptoms on a large group of mothers and their families [6-8], specific evidence-based prevention or intervention programs are still lacking. Although some studies provide evidence for positive effects of postnatal debriefing and counselling after traumatic childbirth [52,53], the effects are only modest. Focusing on established risk factors for PTSD following childbirth, including cognitive variables, may increase the efficacy of interventions in this area. Earlier research has shown that interventions targeting cognitive factors derived from Ehlers and Clark’s theoretical model are not only efficacious for the treatment of chronic PTSD [54], but are also highly effective as an early intervention following trauma [55].

## Conclusions

Established prenatal and birth-related variables explain a substantial amount of variance in PTSD symptoms, but the amount of explained variance can be substantially increased from 43% to a total amount of 68% when theoretically-derived cognitive variables are additionally taken into account. Therefore, the cognitive variables may improve the identification of women who are in need of a therapeutic program to meet PTSD symptoms and they might also be promising targets for specific prevention programs. Future research should investigate cognitive predictors including maladaptive coping strategies in longitudinal studies.

### Endnote

^a^This is a requirement for online studies made by the local ethics committee.

## Competing interests

The authors declare that they have no competing interests.

## Authors’ contributions

TE and CF conceived of the study, and developed the study material. CF carried out data collection. AVE and CF analysed the data, and AVE drafted the manuscript. All authors read and approved the final manuscript.

## Pre-publication history

The pre-publication history for this paper can be accessed here:

http://www.biomedcentral.com/1471-244X/14/200/prepub
